# The loss of microbial autotoxin degradation functions is associated with the decline of beneficial bacterial agents induced by phenolic acids

**DOI:** 10.1128/spectrum.03380-22

**Published:** 2023-09-12

**Authors:** Baoying Wang, Yulan Lin, Wenhao Yu, Qing Xia, Ahmad Ali, Fugang Wei, Chuanchao Dai, Jinbo Zhang, Zucong Cai, Jun Zhao

**Affiliations:** 1 School of Geography, Nanjing Normal University, Nanjing, China; 2 College of Life Sciences, Nanjing Normal University, Nanjing, China; 3 Miaoxiang Sanqi Technology Co., Ltd., Wenshan, China; 4 Jiangsu Engineering Research Center for Soil Utilization & Sustainable Agriculture, Nanjing, China; 5 Jiangsu Center for Collaborative Innovation in Geographical Information Resource Development and Application, Nanjing, China; North-West University, Mmabatho, Mafikeng/North West, Northwest Province, South Africa

**Keywords:** *Panax notoginseng*, Root rot, beneficial agents, autotoxin degradation functions, phenolic acids

## Abstract

Continuous cultivation of medicinal plants can disrupt the rhizosphere’s microbial community. There is still a need to know about the beneficial bacterial community, their putative drivers, and the potential functions they may have. This study used different growth years of Sanqi ginseng (*Panax notoginseng*) with root rot to look at the beneficial microbial community structure, the function of microbial carbon source utilization, and the function of rhizosphere soil metabolism. The beneficial bacterial community changed and the relative abundance of beneficial agents was suppressed significantly with the successive Sanqi ginseng plantings. The carbon source utilization capacity and diversity increased significantly, whereas three autotoxin degradation-related pathways (biosynthesis of other secondary metabolites, metabolism of terpenoids and polyketides, and xenobiotics biodegradation and metabolism) were downregulated considerably with planting year extended. The changes in the beneficial agents were driven by the shifts in phenolic acid profiles, and the decline of beneficial microbes led to the loss of microbial autotoxin degradation functions. Overall, these results provide insight into beneficial microbes, microbial functions, phenolic acids, and their interactions, and these findings are essential for maintaining healthy and sustainable cultivation of Sanqi ginseng.

**IMPORTANCE:**

Sanqi ginseng is a valuable perennial Chinese herb with various benefits for human health. However, continuous cultivation causes a high incidence of root rot disease, which leads to decreased yield and serious economic losses and ultimately impedes the sustainable development of Chinese medicine production. The significance of this study is to reveal the pattern of changes in beneficial bacteria and their related functions in root rot diseased rhizosphere with the successive planting years of Sanqi ginseng. This study found that the decline of beneficial bacterial agents mediated by phenolic acid profiles appears to be associated with the loss of microbial autotoxin degradation functions. This result may have new implications for deciphering the causes of Sanqi ginseng’s continuous cropping obstacles.

## INTRODUCTION

Many crops, especially medicinal plants, suffer significant economic losses as a result of continuous cropping obstacles (1). These obstacles have been linked to a variety of factors, including the buildup of pathogens, disturbances in the soil physicochemical properties, disruptions in the soil microbial communities, and the accumulation of allelochemicals (2). This phenomenon has been identified in many herbal medicines, such as *Panax ginseng* (3), *Rehmannia glutinosa* (1), and *Pseudostellaria heterophylla* (4), and it tends to worsen over time. Sanqi ginseng [*Panax notoginseng* (Burk.) F. H. Chen] is a valuable Chinese herb that is widely cultivated and medicinally utilized due to its therapeutic characteristics, ability to disperse blood stasis, and cardiovascular protective effects (5, 6). In general, Sanqi ginseng is a perennial herbaceous plant that requires to grow for at least 3 years *in situ* before harvest and thrives in a humid, warm, and shaded environment (7, 8). However, the continuous cropping systems and the intensive growing conditions disrupt the soil microbial communities, reducing the number of beneficial bacteria and increasing the abundance of pathogenic microbes, ultimately leading to the prevalence of Sanqi ginseng root rot disease (9). In addition, a previous study reported that the composition and structure of the bacterial community changed significantly over the cropping years of Sanqi ginseng (10). However, how the beneficial bacterial agents shift over the continuous cropping years of root rot-diseased Sanqi ginseng remains unclear.

Disturbances in microbial community structure often led to variations in microbial functions (11). Here, we evaluated microbial carbon source utilization capacity and soil metabolic functions. Soil carbon source utilization capacity is an essential biological characteristic of soil activity and is very sensitive to changes in the soil microbial community (12). A previous study found that the capability and patterns of carbon source utilization changed significantly over the continuous cropping years of healthy Sanqi ginseng (13). In addition, a previous study revealed that the decrease in the abundance of toxic diisobutyl phthalate (DiBP)-degrading microbes led to the accumulation of toxic compounds with the growth of *Panax ginseng* (3). The primary bioactive components in Sanqi ginseng are ginsenosides, which are triterpenoid saponins. However, ginsenosides have been found to be the most significant autotoxin, and the accumulation of autotoxin can ultimately lead to the failure of replanting Sanqi ginseng (14). Ginsenosides can be released into the rhizosphere soil through root secretion, maceration, or plant litter decomposition (15). Although autotoxins accumulate in the rhizosphere with plant growth, the reduced degradation capacity of autotoxin in diseased rhizosphere soils is also a primary reason for the build-up of toxic substances. Therefore, it is necessary to understand how changes in the root-rotten Sanqi ginseng rhizosphere bacterial community, particularly beneficial bacterial agents, affect the microbial functions, mainly the autotoxin degradation.

It is well understood that phenolic acids are thought to be the most important regulators of the soil microbiome (16). In our previous study, we found that the phenolic acid profile was significantly affected by the growth age of root rot-infected Sanqi ginseng, and that the shifts in phenolic acid profiles drive the succession of pathogenic microbiomes. Meanwhile, the amendments of phenolic acids could shift the soil bacterial community structures (17). In particular, the vanillic acid in the cucumber rhizosphere modulated the total bacterial as well as the beneficial *Pseudomonas* and *Bacillus* communities (18). Therefore, we hypothesized that changes in the profile of phenolic acids in the rhizosphere soil of Sanqi ginseng are important drivers of structural and functional changes in the beneficial bacterial communities. The objectives of this study were (i) to investigate the yearly shifts of beneficial bacterial agents and how they relate to phenolic acid profiles and (ii) to disclose how the changes in beneficial microbes influence microbial functions. To address these questions, rhizosphere soil samples of root rot-infected Sanqi ginseng plants at different growth ages were collected. High-throughput sequencing combined with Biolog and Phylogenetic Investigation of Communities by Reconstruction of Unobserved States (PICRUSt) was used to investigate the changes in the structure and function of the bacterial communities and their relationship with phenolic acid profiles.

## RESULTS

### Whole bacterial diversity and composition

**Fig 1:**
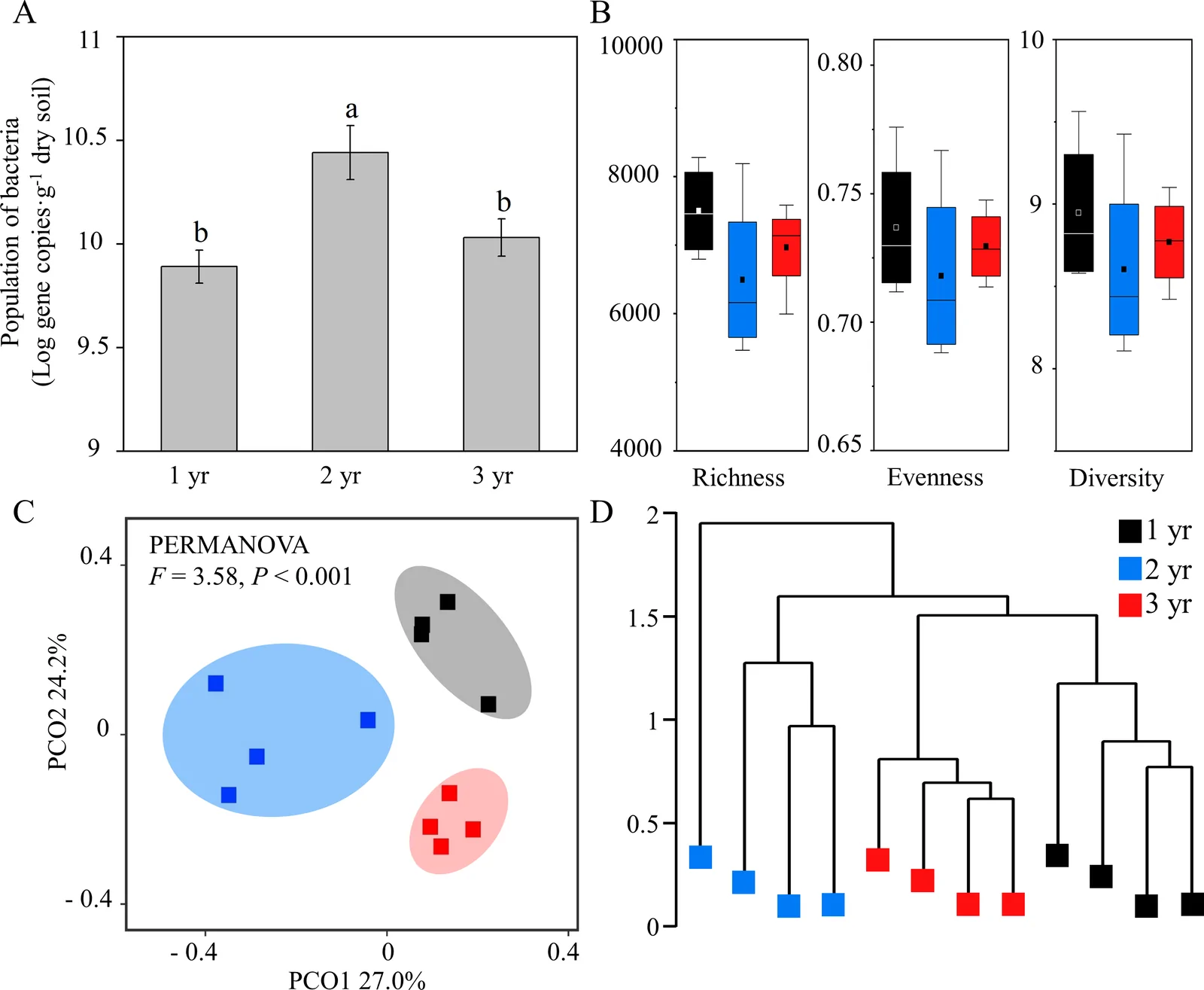
The population of bacteria (**A**) and soil bacterial richness, evenness, and diversity (**B**). Principal coordinates analysis (**C**) and hierarchical cluster analysis (**D**) of the bacterial community based on the Bray-Curtis distance in the soil samples with different plant ages. Error bars indicate the standard errors of the mean of four replicates. Different letters indicate significant differences according to Duncan’s test (*P* < 0.05). The 1 yr, 2 yr, and 3 yr represent the rhizosphere soils that were collected from 1-, 2-, and 3-year-old root rot-diseased Sanqi ginseng plants, respectively.

Over the years of the Sanqi ginseng planting, the bacterium population generally grew and subsequently decreased. Compared to the 1 yr, the rhizospheric soils showed a significant (*P* < 0.05) promotion of bacterial abundance in the 2 yr, followed by suppression in the 3 yr (Fig. 1A). Contrarily, the richness, diversity, and evenness indexes exhibited only a small decreasing tendency from planting years 1 to 2 of Sanqi ginseng and, subsequently, an upward trend in the 3 yr rhizospheric soils (Fig. 1B). Similarly, the principal coordinates analysis (PCoA) ordination plot indicated that the soil bacterial community structure has also changed significantly (permutational multivariate analysis [PERMANOVA], *P* < 0.001) over the Sanqi ginseng planting years (Fig. 1C). In 2 yr rhizospheric soils, the within-group Bray-Curtis distance was considerably (*P <* 0.05) higher than that in 1 yr and 3 yr soils, indicating greater variability among bacterial populations from the same planting year (Fig. S1). Moreover, hierarchical cluster analysis revealed that 1-yr and 3-yr branches were clustered together and differed from 2 yr rhizospheric soils, resulting in the highest Bray-Curtis distance across groups (Fig. 1D; Fig. S1).

**Fig 2:**
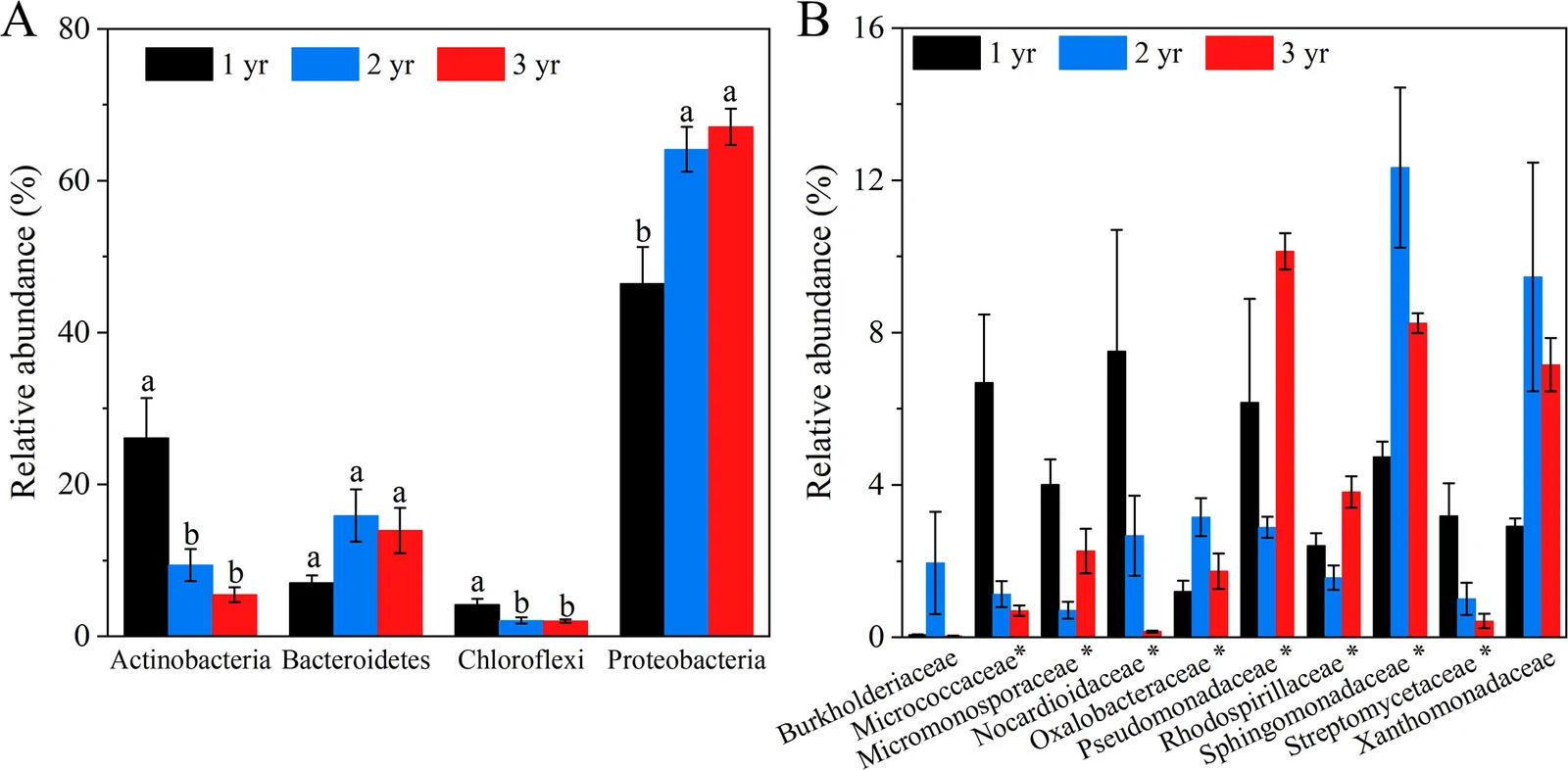
Differences in the relative abundance of the dominant bacterial phyla (**A**) and families (**B**) indicated by LEfSe in the soil samples with different plant ages. Error bars indicate the standard errors of the means of four replicates. Different letters and the asterisk above the taxa represent significantly different according to Duncan’s test (*P* < 0.05).

The results of the linear discriminant analysis effect size (LEfSe) revealed that the root rot-infected Sanqi ginseng rhizosphere soil bacterial community composition significantly changed from phylum to genera levels (Fig. S2). At an linear discriminant analysis (LDA) threshold of 4.0, the cladogram showed that a total of 21 microbial traits exhibited highly variable abundances during the Sanqi ginseng planting years. At the phylum level, the relative abundances of Actinobacteria and Choroflexi were significantly (*P* < 0.05) suppressed, whereas the relative abundance of Proteobacteria was dramatically (*P* < 0.05) increased during the Sanqi ginseng planting years. In addition, the relative abundance of Bacteroidetes increased along with the planting years of Sanqi ginseng, although no significant difference was found in the growth years (Fig. 2A). At the family level, Nocardioidaceae and Streptomycetaceae were significantly (*P* < 0.05) enriched in 1 yr and subsequently depressed over the Sanqi ginseng planting years. The families Oxalobacteraceae, Xanthomonadaceae, and Sphingomonadaceae were more abundant in 2 yr, whereas Rhodospirillaceae and Pseudomonadaceae were significantly (*P* < 0.05) enriched in 3 yr (Fig. 2B).

### Beneficial bacterial diversity and composition

**Fig 3:**
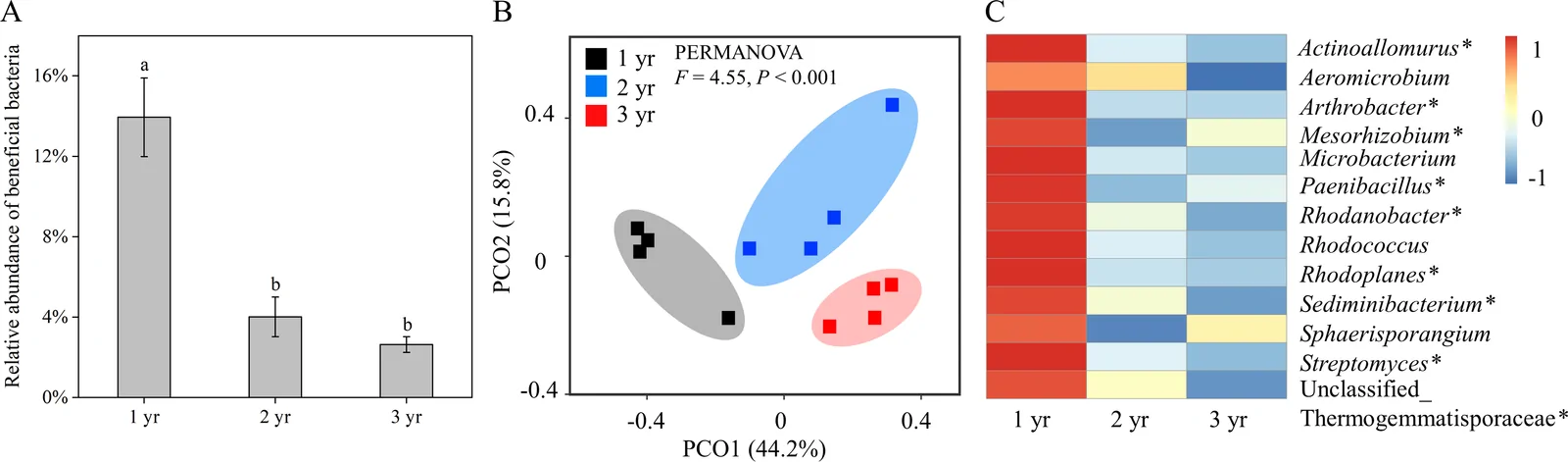
Relative abundance of total beneficial bacterial (**A**) and principal coordinates analysis based on the beneficial bacterial OTUs (**B**) with the Bray-Curtis distance and the relative abundance of beneficial bacteria genera (**C**) in the different plant age soil samples. Error bars indicate the standard errors of the means of four replicates. Different letters and the asterisk above the taxa indicate significant differences at *P* < 0.05 according to Duncan’s tests.

The beneficial bacterial communities were greatly influenced by the cultivation years of Sanqi ginseng. Overall, a total of 13 beneficial genera were identified, with relative abundances accounting for 13.93% in 1 yr, 4.01% in 2 yr, and 2.64% in 3 yr (*P* < 0.001) (Fig. 3A). While evenness and diversity showed a similar tendency, increasing from 1 yr to 2 yr and then declining in 3 yr rhizospheric soils, the richness of beneficial communities significantly (*P* < 0.05) decreased (Table 1). The number of unique beneficial operational taxonomic units (OTUs) in the 1 yr, 2 yr, and 3 yr was 60, 10, and 5, respectively (Table S1). These data suggest that as planting years increased, the diversity of the unique beneficial bacteria drastically decreased. There were 53 core beneficial OTUs, which represented, respectively, 37.1, 63.1, and 74.7% of all retained beneficial OTUs and 94.3, 90.5, and 94.9% of all beneficial sequences of 1 yr, 2 yr, and 3 yr (Table S1). Additionally, the community structure of beneficial microbes altered dramatically during the Sanqi ginseng planting years (PERMANOVA, *P* < 0.001) (Fig. 3B). Specifically, the beneficial bacterial communities clearly displayed a decreasing pattern in the relative abundance data, as seen by the heatmap illustration (Fig. 3C). In particular, 1 yr rhizosphere soils had considerably (*P* < 0.05) greater relative abundances of genera *Actinoallomurus*, *Arthrobacter*, *Paenibacillus*, *Rhodoplanes*, and *Streptomyces* compared to 2 yr and 3 yr.

**TABLE 1:** Richness, Shannon diversity, and evenness of beneficial soil bacterial communities derived from different planting years of root rot-infected Sanqi ginseng^*a*^

Planting year	Beneficial bacteria		
	Richness	Diversity	Evenness
1 yr	226.8 ± 15.0a	3.84 ± 0.23	0.49 ± 0.04b
2 yr	142.8 ± 10.7b	4.81 ± 0.28	0.67 ± 0.05a
3 yr	112.5 ± 8.7b	4.35 ± 0.29	0.64 ± 0.03a

^*a*^ Values (means ± SE, *n* = 4) within the same column followed by different letters are significantly different at *P* < 0.05 according to Duncan’s tests.

### Substrate utilization pattern and functional diversity

**Fig 4:**
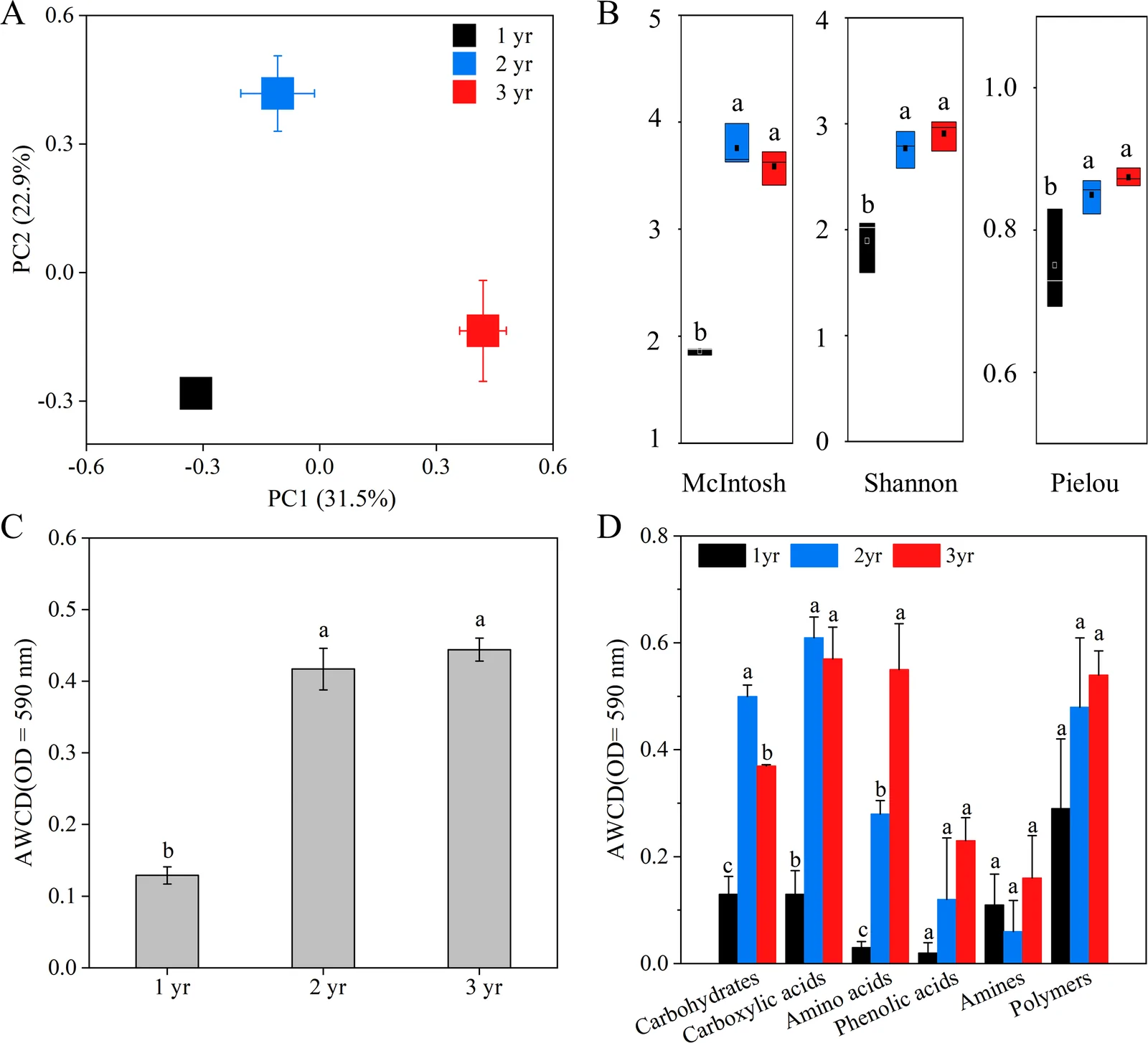
The principal component analysis (**A**) and soil microbial metabolic richness, diversity, and evenness (**B**), average well color development (**C**), and categorized carbon utilization pattern (**D**) calculated from the Biolog data after a 6-day incubation. Error bars indicate the standard errors of the means of four replicates. Different letters represent significantly different at *P* < 0.05 according to Duncan’s tests.

In general, the substrate utilization patterns of root rot-infected Sanqi ginseng rhizosphere soils were significantly (PERMANOVA, *P* < 0.05) altered over the course of planting years (Fig. 4A). The difference in the utilization of carbon sources across treatments was explained by the first two principal components, with respective variances of 31.5% and 22.9%. Additionally, the substrate utilization diversity and evenness were significantly (*P* < 0.05) affected by Sanqi ginseng planting years. The McIntosh and Shannon diversity and Pielou evenness were significantly (*P* < 0.05) higher in 2 yr and 3 yr than in 1 yr rhizospheric soils (Fig. 4B).

The average well color development (AWCD) values for 1 yr rhizospheric soils indicated a slight increase with extended incubation time, but they remained at a low level (Fig. S3). Additionally, the AWCD values increased significantly over the Sanqi ginseng planting years and were evidently (*P* < 0.05) higher in 2-yr and 3-yr rhizospheric soils. However, there is no significant difference between 2-yr and 3-yr rhizospheric soils (Fig. 4C). All six categorized carbon substrates had weak metabolism in 1 yr rhizospheric soils after 6 days of incubation. Furthermore, the utilization capabilities of carbohydrates, carboxylic acids, and amino acids were significantly increased, while the utilization of phenolic acids, amines, and polymers changed insignificantly with Sanqi ginseng planting years (Fig. 4D). These findings suggest that the Sanqi ginseng planting years were accompanied by an improvement in the microbial metabolic activity and the ability to utilize various single carbon sources.

### The microbial metabolism functions predicted by PICRUSt

**Fig 5:**
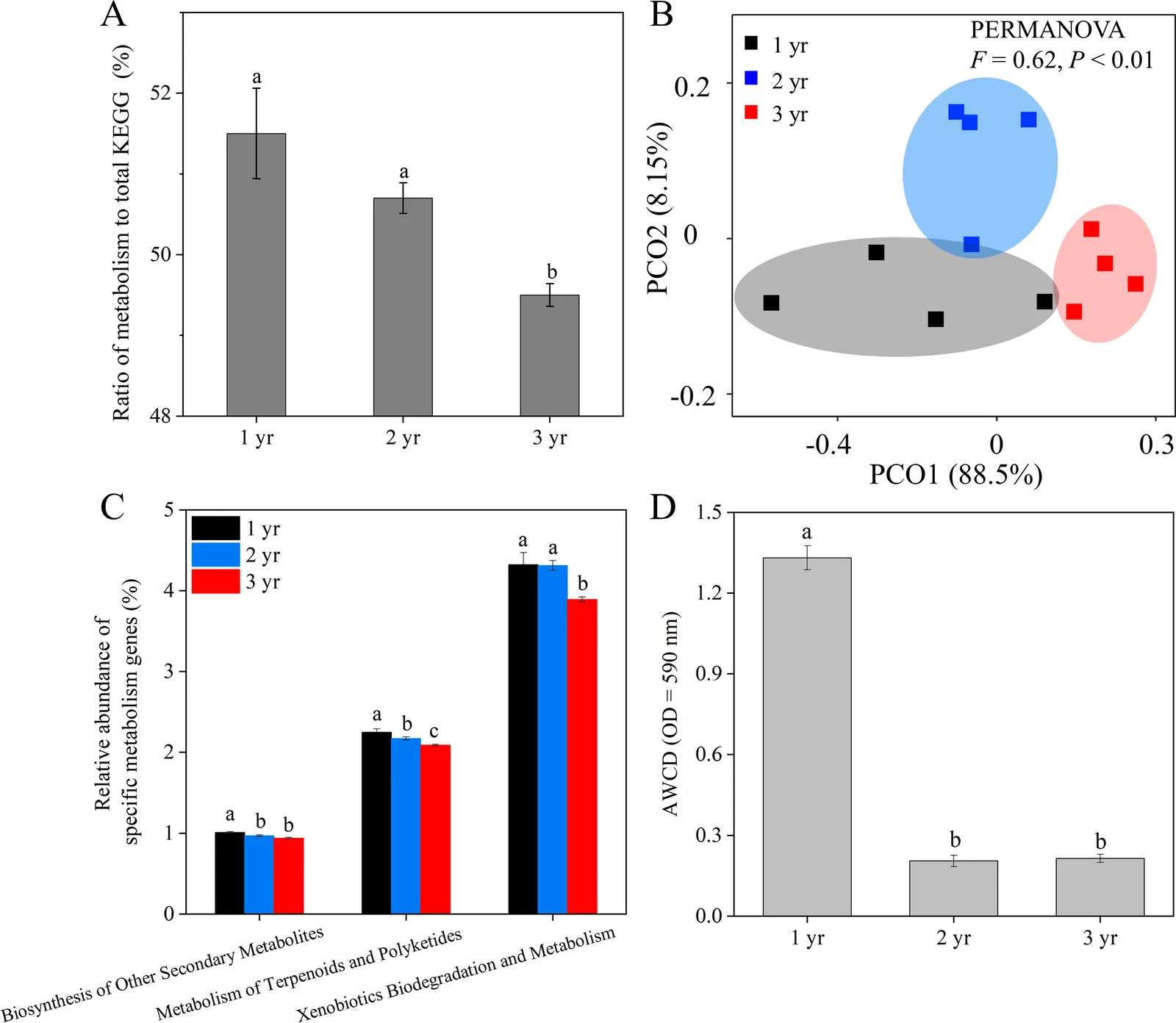
Relative abundance of metabolism pathways (**A**) and principal coordinates analysis of all metabolism genes (**B**) and relative abundance of autotoxin degradation genes: the biosynthesis of other secondary metabolites, metabolism of terpenoids and polyketides, and xenobiotics biodegradation and metabolism in different plant year soils (**C**) and the average well color development of the ginsenoside utilization. Error bars indicate the standard errors of the means of four replicates. Different letters represent significantly different at *P* < 0.05 according to Duncan’s tests.

We used PICRUSt to predict the bacterial metabolic activities to investigate the impact of planting years on root rot-infected Sanqi ginseng rhizospheric soil function. Overall, six categories of biological metabolic pathways (level 1) were identified. These were organismal systems, metabolism, human diseases, genetic information processing, environmental information processing, and cellular processes (Fig. S4). Among these pathways, metabolism, environmental information processing, and genetic information processing were the primary components, accounting for 49.47%–51.51%, 13.89%–14.30%, and 15.01%–15.26%, respectively. Furthermore, Sanqi ginseng planting years also resulted in a significant improvement in the relative prevalence of human diseases and cellular processes. Among them, metabolism was the dominant pathway, but the relative abundance decreased significantly (*P* < 0.05) with Sanqi ginseng planting years (Fig. 5A).

The PCoA based on the metabolic pathway genes revealed that the Sanqi planting years significantly (PERMANOVA, *P* < 0.01) altered the bacterial metabolism function (Fig. 5B). Furthermore, we focused on some metabolic pathways involved in the degradation of autotoxin (level 2). In particular, as planting years increased, the relative abundance of three specific genes linked to the degradation of autotoxin, such as those involved in the biosynthesis of other secondary metabolites, the metabolism of terpenoids and polyketides, and the biodegradation and metabolism of xenobiotics, drastically decreased (*P* < 0.05) (Fig. 5C). It was then confirmed by ginsenoside utilization validation experiments, showing that the ginsenoside utilization capacity of diseased Sanqi ginseng plants decreased with the increase in Sanqi ginseng cultivation. And the utilization capacity was highest in 1 yr and then decreased significantly (*P* < 0.05) in 2 yr and 3 yr (Fig. 5D). These results suggested that the microbial autotoxin degradation functions in the rhizosphere of Sanqi ginseng were gradually depleted with growth.

### Correlations between the beneficial bacterial communities and autotoxin degradation functions

In general, we found a positive correlation between the activity and diversity of carbon substrate utilization and the abundance of bacteria. Particularly, the McIntosh diversity revealed significant (*P* < 0.05) relationships with the abundance of bacteria. However, there were dramatically negative correlations (*P* < 0.05) between the diversity and activity of carbon substrate utilization and the relative abundance of beneficial bacteria (Table S2).

**Fig 6:**
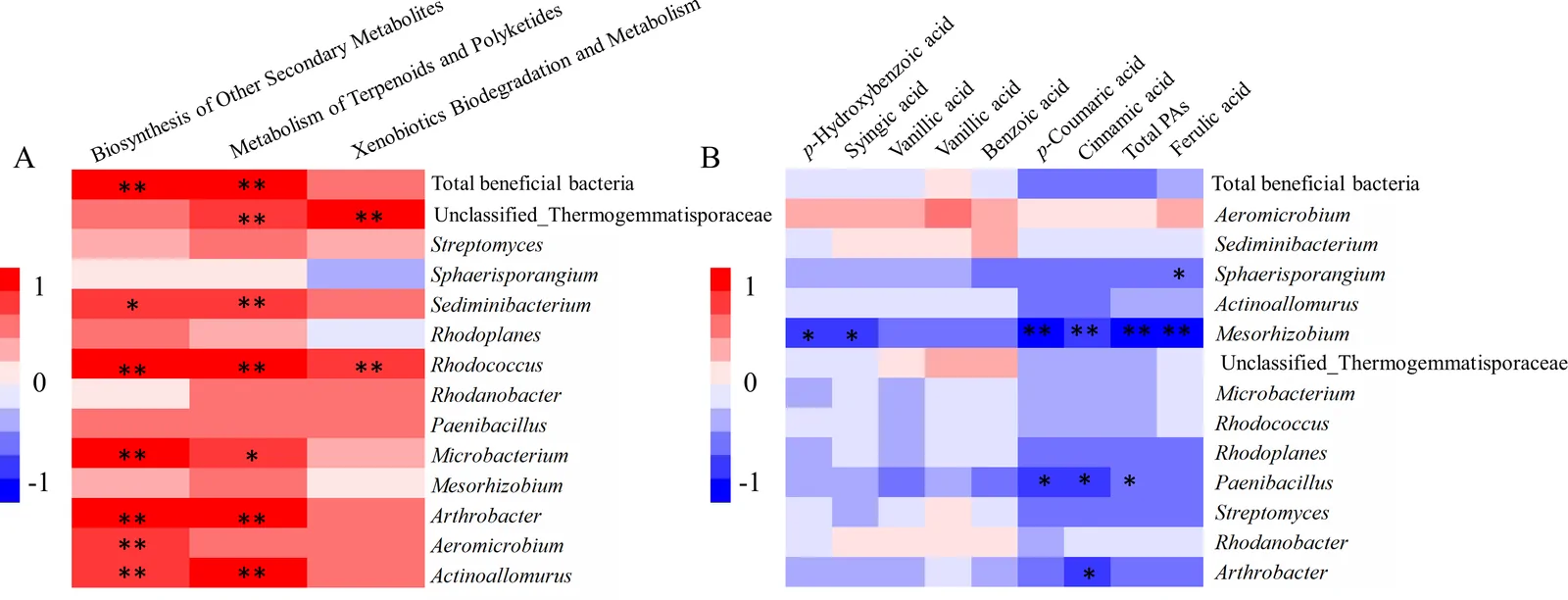
Spearman’s rank-order correlation between the relative abundance of beneficial bacterial genus and autotoxin degradation genes (**A**) and phenolic acids content and relative abundance of the beneficial bacteria at the genus level. The key from blue to red represents the negative correlation to the positive correlation. *P < 0.05; **P < 0.01.

In particular, the relative abundance of the beneficial bacteria showed positive correlations with the relative abundance of three metabolic genes related to autotoxin degradation. For example, the relative abundance of the beneficial bacteria genera, including *Actinoallomurus*, *Arthrobacter*, *Microbacterium, Sediminibacterium*, and the total beneficial bacterium was significantly and positively correlated with the relative abundances of the biosynthesis of other secondary metabolites and the metabolism of terpenoids and polyketides. In addition, the relative abundance of *Rhodococcus* and unclassified_Thermogemmatisporaceae showed a significant (*P* < 0.05) and positive correlation with the genes of xenobiotics biodegradation and metabolism (Fig. 6A).

### Linking the beneficial communities to the phenolic acid profiles

The Procrustes analysis revealed a significant correlation between the ordination of phenolic acid profiles and both the bacterial communities (*M* ^2^ = 0.41, *P* < 0.001) and the beneficial communities (*M* ^2^ = 0.56, *P* < 0.01) (Fig. S5A and B). In addition, the α-diversity of the beneficial communities was significantly affected by the phenolic acid profiles (Table 2). For instance, the Shannon diversity and evenness of the phenolic acid had significant (*P* < 0.05) and positive correlation with the richness of the beneficial bacterium. Interestingly, all the phenolic acid content was positively correlated with the Shannon diversity and evenness of beneficial bacterium. In particular, the contents of syringic acid, cinnamic acid, and total phenolic acid showed a significant (*P* < 0.05) and positive correlation with the Shannon diversity and evenness of beneficial bacterium.

**TABLE 2:** Spearman’s rank-order correlations between phenolic acids and α-diversity of beneficial communities calculated from the OTU data

Phenolic acid	Beneficial bacteria		
	Richness	Diversity	Evenness
Shannon	**0.69** ^*a*^	−0.34	−0.40
Evenness	**0.69** ^*a*^	−0.34	−0.40
*p*-Hydroxybenzoic acid	−0.04	0.54	0.49
Vanillic acid	0.15	0.38	0.30
Syringic acid	−0.04	**0.63** ^*a*^	0.57
Vanillin	0.05	0.43	0.39
*p*-Coumaric acid	−0.45	0.55	0.55
Ferulic acid	−0.34	0.53	0.50
Benzoic acid	−0.07	0.41	0.36
Cinnamic acid	−0.25	**0.65** ^*a*^	**0.64** ^*a*^
Total PAs	−0.31	**0.62** ^*a*^	**0.60** ^*a*^
			

^*a*^ Values in bold indicate significant correlations, *P < 0.05.

Predominantly, the relative abundance of genera *Arthrobacter*, *Paenibacillus*, *Rhodoplanes*, *Rhodococcus*, *Microbacterium*, *Mesorhizobium*, *Actinoallomurus*, and *Sphaerisporangium* showed a negative correlation with the contents of all individual and total phenolic acids. In particular, the relative abundance of the genus *Arthrobacter* was significantly (*P* < 0.05) and negatively correlated with the content of cinnamic acid. The relative abundance of genera *Paenibacillus* and *Mesorhizobium* showed significantly (*P* < 0.05) negative correlations with the content of *p*-coumaric acid, cinnamic acid, as well as the total phenolic acids. Interestingly, there was a negative correlation between the relative abundance of total beneficial bacterium and the content of total phenolic acids (Fig. 6B).

## DISCUSSION

### Root rot-infected Sanqi ginseng shapes a yearly decline of beneficial bacterial agents by regulating phenolic acid profiles

Bacteria are major members of soil microbiota and the rhizosphere bacterial community has a vital role in promoting plant growth and health (19). In the present study, we observed significant differences in the composition and structure of rhizosphere bacterial communities of Sanqi affected by root rot disease at different growth ages. This finding is consistent with a previous study that demonstrated the influence of the growth stage on the rhizosphere bacterial community of Sanqi (10). Further analysis of the bacterial communities at the family level demonstrated that the relative abundance of Nocardioidaceae and Streptomycetaceae belonging to Actinobacteria was significantly suppressed with the growth of Sanqi. Many members of these two families have been widely reported for their ability to control plant pathogens and promote growth (20, 21). In addition, we identified microbes associated with functions such as suppression of soil-borne diseases, biodegradation of toxic substances, efficient nutrient acquisition, and plant growth promotion as beneficial agents. In this study, we observed that the relative abundance of beneficial genera and total relative abundance also decreased significantly with the growth of Sanqi ginseng. This result was in conformity with a recent study, which suggested that the re-assembly of the rhizosphere microbiome with the growth of Sanqi ginseng could suppress some beneficial antagonistic bacteria, such as *Pseudomonas* and *Bacillus*. Similar findings were also reported in ginseng rhizosphere (22). Moreover, *Arthrobacter* and *Streptomyces* were the most abundant beneficial bacteria in the Sanqi ginseng rhizosphere, and some species of these genera are potential biological control agents for Sanqi root rot disease caused by dominant pathogens like *Fusarium oxysporum*, *Fusarium solani* (23), and *Cylindrocarpon destructans* (24). The resultant depletion of these beneficial agents may lead to the proliferation of pathogenic microbes in the consecutive planting system.

Previous studies have demonstrated that phenolic acids are the pivotal component of root exudates that might mediate the succession of rhizosphere bacterial communities (25, 26). In particular, mounting evidence demonstrates that phenolic acids drive changes in rhizospheric beneficial bacterial communities. For instance, the composition and structure of the *Bacillus* communities and the beneficial *Pseudomonas* in the cucumber rhizosphere were significantly impacted by vanillic acid (27). Procrustes analysis revealed a substantial correlation between the phenolic acid profiles and the overall bacterial communities as well as the beneficial bacterial communities specifically. Moreover, the diversity and evenness of the phenolic acids showed a significant correlation with the richness of the beneficial community. This indicates that phenolic acid profiles may be a key factor driving the succession of the beneficial bacterial communities in the rhizosphere soil of root rot-infected Sanqi. Furthermore, the Spearman’s correlation analysis suggested that almost all the phenolic acids negatively affected all beneficial bacterium other than *Aeromicrobium*. Specifically, the most abundant beneficial genera *Arthrobacter* and *Streptomyces* showed negative correlations with all the phenolic acids except vanillic acid. This is in accordance with a previous observation, which demonstrated that syringic acid and benzoic acid negatively correlated with *Streptomyces* in the *Radix pseudostellariae* rhizosphere (28). Additionally, a recent culture study of Sanqi has validated that *p*-hydroxybenzoic acid, ferulic acid, vanillic acid, and a mixture of five phenols could suppress the growth and biofilm formation of beneficial bacteria (*Burkholderia* sp. and *Lysobacter* sp.) (29). These results imply that root rot-infected Sanqi ginseng could shape the yearly decline of beneficial bacterial agents by modulating the phenolic acid profiles.

### Decline of beneficial microbes leads to the loss of microbial autotoxin degradation functions, not carbon source utilization function

Soil metabolic activity and diversity are important biological indicators of soil ecological functions (12). In the current study, the carbon source utilization pattern changed significantly with the growth years of Sanqi ginseng. This pattern was consistent with several earlier studies that the soil microbial metabolic pattern significantly changed with the planting years of many crops. Such dramatic changes are mainly due to the yearly shifts in the structure and composition of soil microbial communities (30, 31). Moreover, in line with a previous finding, we observed that the metabolic activity and diversity significantly increased along with the planting years of Sanqi (32). This may be because the carbon utilization characteristics were mainly dependent on the abundance, diversity, and activity of the microbial community. Further Spearman’s correlation analysis revealed that the activity and diversity of carbon utilization were significantly and positively associated with the abundance of bacteria while negatively related to the relative abundance of the beneficial bacteria. It is therefore plausible that the decline of the beneficial bacteria did not cause the decrease of carbon source utilization function.

PICRUSt analysis was applied to estimate the functional potentials of the microbial communities in the rhizosphere of Sanqi ginseng. The results revealed that the predicted functions varied significantly throughout the growth years. This study found metabolism pathways to be the most dominant microbial functions in Sanqi ginseng rhizosphere soils. This supports a previous finding that metabolic pathways play a crucial role in the ecological function of Sanqi ginseng soil (33). However, the relative abundance of metabolism genes decreased significantly with increasing Sanqi cultivation years. Particularly, three autotoxin degradation genes were downregulated when planting years were extended, as they were involved in the biosynthesis of other secondary metabolites, the metabolism of terpenoids and polyketides, and xenobiotics biodegradation and metabolism. Among them, secondary metabolites were reported to be highly enriched in resistant Sanqi varieties and were linked to the disease-suppressive function of Sanqi ginseng (34). It was reported that xenobiotics biodegradation and metabolic pathways were crucial for the biodegradation of organic pollutants and pesticide residues (35). Moreover, it was shown that the biosynthesis or degradation of ginsenoside in Sanqi ginseng is associated to the metabolism of terpenoids and polyketides pathways (36). We further found that these functional abilities decline due to the decreased relative abundance of the beneficial agents. These beneficial groups have been found to exert a variety of helpful effects, such as producing antibiotics and degrading toxic substances to sustain host-plant health (33). For instance, a member of Actinobacteria known as *Rhodococcus* could produce some secondary bioactive compounds to suppress soil-borne diseases, and some species of *Rhodanobacter* and *Rhodoplanes* may be responsible for the biodegradation of pesticides (37, 38).

Ginsenosides, which belong to the class of terpenoids, are the main active ingredient of Sanqi. They can be released by Sanqi residues and root exudates and accumulate in the soil with the growth of Sanqi and may be autotoxic to seedlings or promote the growth of soil-borne pathogens (9, 14). A recent study suggests that the decrease in the degradation capacity of autotoxic in the rhizosphere soil may be one of the mechanisms of disease occurrence (39). In our study, the relative abundance of the metabolism genes of terpenoids and polyketides depressed with Sanqi ginseng cultivation, which was then verified by crude ginsenoside utilization tests. Similar results were found in studies on *Panax ginseng*, where the abundance of DiBP-degrading microbes declined continuously with ginseng cultivation leading to the accumulation of toxic compounds (2). In the present study, many beneficial bacteria showed positive correlations with the metabolism of terpenoids and polyketides. This is in line with a previous study, which revealed that *Streptomyces* and *Paenibacillus* may be involved in the biodegradation of ginsenosides Rb1 and Rh1, thus indicating that some beneficial microbes are important for the elimination of autotoxin (9). Thus, ginsenoside accumulation in the Sanqi rhizosphere may be due to decreasing specific bacterial degradability as plant age increased.

### Conclusion

In conclusion, our findings showed that the growth ages of root rot-infected Sanqi ginseng significantly influenced the rhizospheric beneficial community, carbon source utilization capacity, and metabolism functions. Specifically, during the growth of Sanqi ginseng, the beneficial bacterial community structure changed significantly, and the relative abundance of beneficial microbes decreased notably due to the modulation of phenolic acids. In addition, reducing of beneficial microbes caused a loss of microbial autotoxin degradation functions rather than carbon source utilization functions. The findings of this study might offer new perspectives on the problem of resolving the replanted crop disease that affects Sanqi ginseng.

## MATERIALS AND METHODS

### Field study site and soil sampling

The field study was performed at Miaoxiang Sanqi Technology Co., Ltd., located in Wenshan County, Yunnan Province of southwestern China (23°42´ N, 104°16´ E), which is the leading region of Sanqi production. The area has a typical subtropical monsoon climate with an average annual temperature and rainfall of 16°C and 1,200 mm, respectively. The mean annual sunshine hours are 1,500–2,000 h, and the soil is classified as yellow-brown.

The experiment was conducted in one of the Sanqi ginseng gardens, which had not previously been planted with Sanqi ginseng. The specific experimental design has been described in detail previously, and the physicochemical properties of the soil are shown in Table S3 (40). Sanqi ginseng seedlings have been continuously cultivated for 1, 2, and 3 years in 2019. Thus, soil samples from the rhizosphere were taken from Sanqi ginseng plants in the early stages of root rot and had been growing for 1, 2, or 3 years (defined as 1 yr, 2 yr, and 3 yr). In brief, 36 root rot-infected Sanqi seedlings of 1 yr, 2 yr, and 3 yr were randomly selected from the plantation. Rhizosphere soil was collected by carefully brushing the soil adhering to Sanqi roots, and three sub-replicate soil samples were pooled together as one sample, and then four rhizosphere soil samples (biological replicates) of each planting year were obtained. The obtained soil samples were then homogenized by passing through a 2-mm sieve and processed as appropriate for microbial functional diversity assays with Biolog and DNA extraction for bacterial abundance and community analyses.

### Soil DNA extraction and bacterial quantification

According to the manufacturer’s instructions, total soil DNA was extracted from 0.5 g of fresh soil using a FastDNA SPIN Kit for Soil (MP Biomedicals, Solon, Ohio, USA). The final DNA concentration and purification were determined by a Nanodrop ND-1000 spectrophotometer (Thermo Scientific, Waltman, MA, USA). The extracted DNA samples were subsequently stored at −20°C for further use.

Real-time PCR was conducted on a CFX-96 thermocycler (Bio-Rad Laboratories Inc., CA, USA) using SYBR Premix Ex Taq (TaKaRa, Dalian, China) to determine the abundance of bacterial 16S rRNA genes, with the primer sets Eub338-CCTACGGGAGGCAGCAG (41) and Eub518-ATTACCGCGGCTGCTGG (42). The real-time PCR reaction mixture for the 16S gene was set according to previous work (43). The standard curve was generated as followed by a previous report (44), and the amplification efficiency for bacteria was 100.9%. The specificity of PCR amplification was assessed by melting curve analysis, and the copy numbers were log_10_-transformed before statistical analysis.

### Illumina miseq sequencing and data processing

Illumina Miseq sequencing was used to discriminate the yearly shifts of bacterial diversity, and community structure of root rot-infected Sanqi ginseng rhizosphere soils. The bacterial 16S rDNA V4–V5 region in the rhizospheric soil DNA samples was amplified using the primer sets 515F (5´-GTGCCAGCMGCCGCGGTAA-3´) and 907R (5´-CCGTCAATTCMTTTRAGTTT-3´) (45). The PCR amplification and thermal conditions were according to a previous study (43). After PCR application, the purified amplicons were pooled and sequenced using a Miseq platform (Illumina, CA, USA) conducted by Major Biotechnologies, Inc. (Shanghai, China). The raw reads were deposited into the NCBI Sequence Read Archive database (accession number: SRP390692).

The Quantitative Insights into Microbial Ecology software package, version 1.9.1 was used to carry out sequence analyses (46). Briefly, the paired-end reads were merged, quality controlled, and clustered into OTUs with a 97% similarity cut-off using Greengenes 13_8 database (47). Finally, the sequences were rarefied to 47, 000 sequences to equalize the sampling effort. Then, the most abundant Kyoto Encyclopedia of Genes and Genomes Ortholog functional profiles reveal the metabolism pathways that were performed based on the OTU table with the PICRUSt approach (48).

### Microbial carbon source utilization functional diversity assay

Soil microbial functional diversity based on their carbon source utilization patterns was estimated by Biolog EcoPlates (Biolog, Inc., Hayward, CA, USA) and an automated microplate reader (BioTek Instruments, Inc., USA). In brief, 5 g of fresh soil sample was mixed with 45 mL of 0.85% sterile NaCl solution and shaken for 30 min at 25°C (220 rpm). Following 10-fold serial dilutions to 10^−3^ and 125 µL of supernatant being injected into each well of the microplates, the samples were run after standing for 30 min. After that, the plates were incubated for 6 days at 25°C in the dark, and every 24 h for a total of 144 h, the optical density (OD) at 590 nm was recorded (12). The OD values were adjusted by subtracting the results from control wells, and the negative values were set to 0 before the additional analysis.

### Biolog data analysis

The metabolic activity of the microbial community was assessed using the AWCD of all substrates, which was computed according to a previous report (49). A total of 31 carbon sources were categorized into six major groups based on their chemical natures, including carbohydrates (*n* = 10), carboxylic acids (*n* = 7), amino acids (*n* = 6), phenolic acids (*n* = 2), amines (*n* = 2), and polymers (*n* = 4). The substrate AWCD values on 144 h were calculated according to previous research (50). The effect of inoculum density on substrate oxidation was normalized and countered by dividing the AWCD values by the absorbance values of each substrate well over a period of 144 h (51). The principal component analysis was used to compare the community consumption patterns in carbon sources along with the planting years of Sanqi. Based on the absorbance values over 144 h, the diversity of microbial metabolism was calculated, with OD >0.2 being regarded as a positive response. This diversity was graded as substrate richness (McIntosh), substrate diversity (Shannon), and substrate evenness (Pielou) (52).

### Evaluation of the ginsenoside utilization

The soil microbial ginsenoside utilization was estimated using a self-made microplate containing the crude ginsenosides. In brief, 20 µL of crude ginsenosides was added to each well of a 96-well sterile microplate as the carbon source, and 5 µL of tetrazolium violet was added as the stain, followed by 125 µL of soil suspension, and the soil suspensions were prepared as before. The microplate was then incubated for 72 h at 170 rpm/min in a shaker at 30°C and the OD values of each well were measured at 590 nm. The crude ginsenosides were extracted from the Sanqi ginseng root tuber according to a previous study (14). In short, 50 g of Sanqi ginseng powder was suspended using 200 mL of 100% MeOH, and the suspension was shaken on a shaker at 180 rpm at 30°C for 2 h and then subjected to ultrasonic extraction at 30°C for 2 h. The powder extract was then centrifuged at 7,000 rpm for 3 min, and the supernatant was concentrated at 30°C using a vacuum rotary evaporator. The dry extracts obtained were then redissolved in 20 mL sterilized water, filtered using a 0.22 µm nylon syringe filter, and kept at 4°C. The crude ginsenosides contained R_1_, Rg_1_, Rb_1_, Rh_1_, and Rd, and the final concentration was 10 mg/mL, 5 mg/mL, 30 mg/mL, 1 mg/mL, and 9 mg/mL, respectively.

### Bioinformatics and statistical analysis

For the statistical analysis, SPSS 19.0 software (SPSS Inc., Chicago, IL) used one-way analysis of variance. The Duncan’s multiple range test (*P* < 0.05) was used to determine the differences between soil samples that were statistically significant. At a depth of 47,000 sequences per sample, bacterial diversity (Shannon), richness (Chao1), and evenness (Pielou) were computed using the rarefied OTU table. PCoA and hierarchical cluster analysis were used to investigate the differences in bacterial community structure based on Bray-Curtis distances.

Potentially beneficial bacteria were predicted according to existing literature. We recognized the microbes associated with functions such as suppression of soil-borne diseases, biodegradation of toxic substances, efficient nutrient acquisition, and promotion of plant growth as beneficial agents. This study identified 13 different bacteria genera as potentially beneficial agents, including *Actinoallomurus*, *Aeromicrobium* (53), *Arthrobacter*, *Mesorhizobium*, *Microbacterium*, *Paenibacillus*, *Rhodococcus* (54), *Rhodanobacter, Rhodoplanes, Sediminibacterium* (55), *Sphaerisporangium, Streptomyces,* and unclassified_Thermogemmatisporaceae (56). The beneficial genera and their corresponding OTU tables were retained for further analysis. The α- and β-diversity of beneficial communities were analyzed based on the beneficial OTU table. The OTUs that consistently appeared in at least three biological replicates for each planting year served as the basis for the analysis of the core and unique beneficial bacteria. LEfSe (http://huttenhower.sph.harvard.edu/galaxy) was applied to identify the microbial taxonomic differences (from phylum to genus) among soil samples. PERMANOVA based on Bray-Curtis index matrices was performed to evaluate the statistical significance of the bacterial and beneficial bacterial and the metabolism pathway gene community differences.

The “procrustes” function in the “vegan” package was used to do the Procrustes analysis between beneficial bacterial communities and the phenolic acid profiles. The phenolic acid data refer to our previous published study (40). To reveal the relationships between the relative abundance of beneficial bacteria and microbial functions, Spearman’s rank correlations were used to examine the relationships between the relative abundance of beneficial bacteria, the carbon source metabolic activity and diversity, and the autotoxin degradation genes.

## Data Availability

The raw reads in this study have been deposited into the NCBI Sequence Read Archive (SRA) database under accession number SRP390692.

## SUPPLEMENTAL MATERIAL

Supplemental Material spectrum.03380-22-s0001.docx Fig. S1-S5; Tables S1-S3.

ASM does not own the copyrights to Supplemental Material that may be linked to, or accessed through, an article. The authors have granted ASM a non-exclusive, world-wide license to publish the Supplemental Material files. Please contact the corresponding author directly for reuse.
