# Supplementary material for: The loss of microbial autotoxin degradation functions is associated with the decline of beneficial bacterial agents induced by phenolic acids
Source: Microbiol Spectr. 2023 Sep 12;11(5):e03380-22. doi: 10.1128/spectrum.03380-22 (PMC10581185; doi:10.1128/spectrum.03380-22)
Supplement: Supplemental Material — Fig. S1-S5; Tables S1-S3. [file spectrum.03380-22-s0001.docx]

**The loss of microbial autotoxin degradation functions is associated with the decline of beneficial bacterial agents induced by phenolic acids**

Baoying Wang^1,2^, Yulan Lin^1^, Wenhao Yu^1^, Qing Xia^1^, Ahmad Ali^1^, Fugang Wei^3^, Chuanchao Dai^2^, Jinbo Zhang^1,4^, Zucong Cai^1, 4,5^, Jun Zhao^1, 4,5^*

^1^School of Geography, Nanjing Normal University, Nanjing, China

^2^College of Life Sciences, Nanjing Normal University, Nanjing, China

^3^Miaoxiang Sanqi Technology Co., Ltd., Wenshan, China

^4^Jiangsu Engineering Research Center for Soil Utilization & Sustainable Agriculture, Nanjing, China

^5^Jiangsu Center for Collaborative Innovation in Geographical Information Resource Development and Application, Nanjing, China

**Running title**: Root rot reduces autotoxin degradation functions of rhizosphere microbiome

*Corresponding author: Jun Zhao

Address: School of Geography, Nanjing Normal University, Nanjing, 210023, China

Tel.: +86-25-85891203, Fax: +86-25-85891745; E-mail: junzhao37@njnu.edu.cn

**
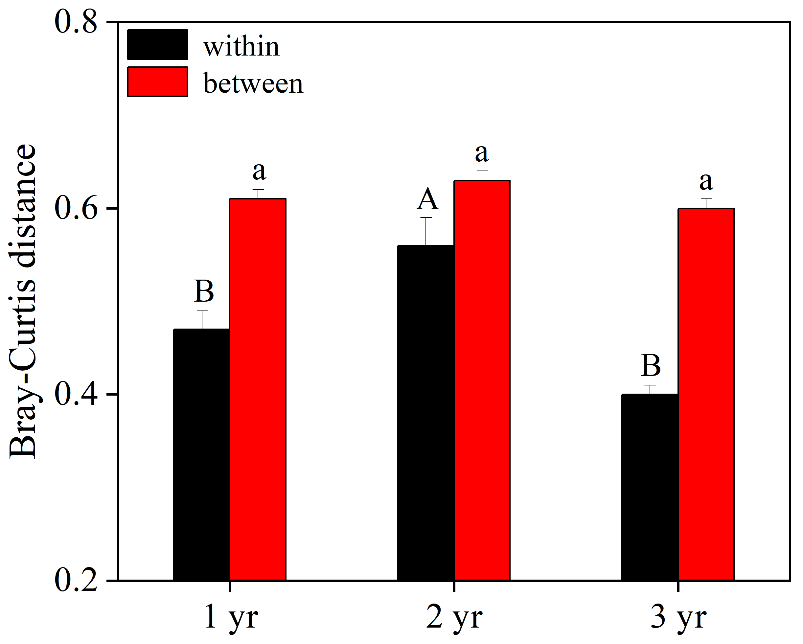
**

**Fig. S1.** The within and between Bray-Curtis distance in the soil samples derived from different planting years.

*Error bars* indicate the standard errors of the means of four replicates. *Different letters* represent significantly different at *p* < 0.05 according to the Duncan’s tests.


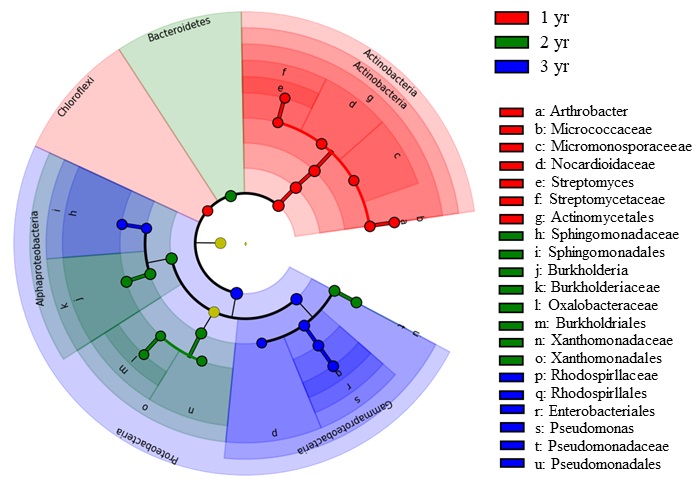


**Fig. S2.** Taxonomic differences of the microbiota from phylum to genera in different treatments. The cladogram was performed by LDA Effect Size (LEfSe) analysis with an online tool (<http://huttenhower.sph.harvard.edu/galaxy>). The alpha value for the factorial Kruskal-Wallis test was 0.05, and the LDA score threshold was 4.0.


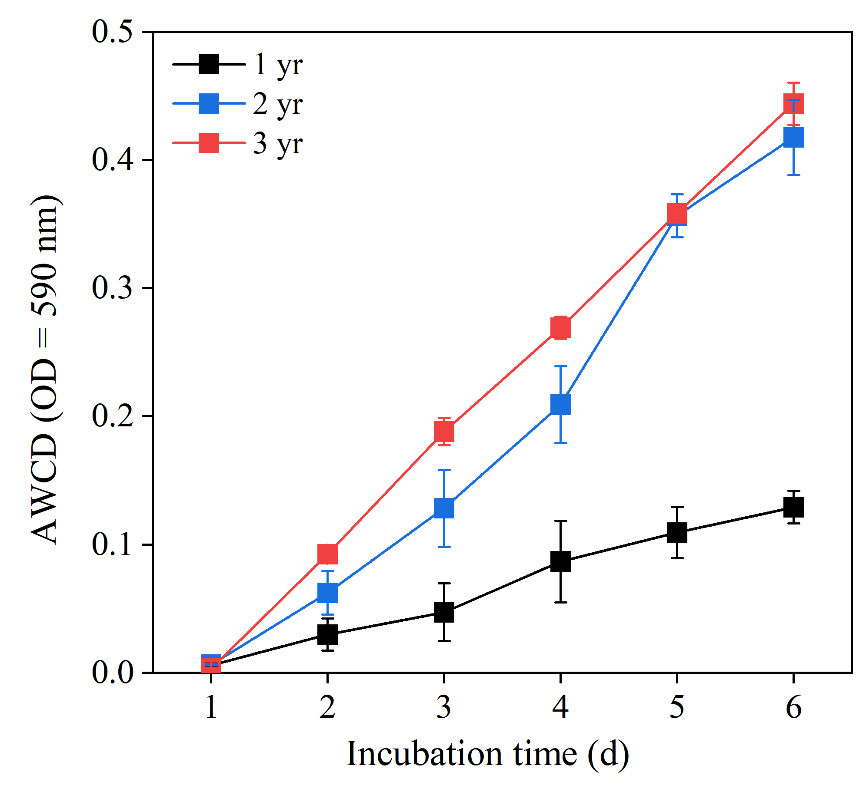


**Fig. S3.** The time course change of average well color development (AWCD) for different treatments. *Error bars* indicate the standard errors of the means of four replicates.

**
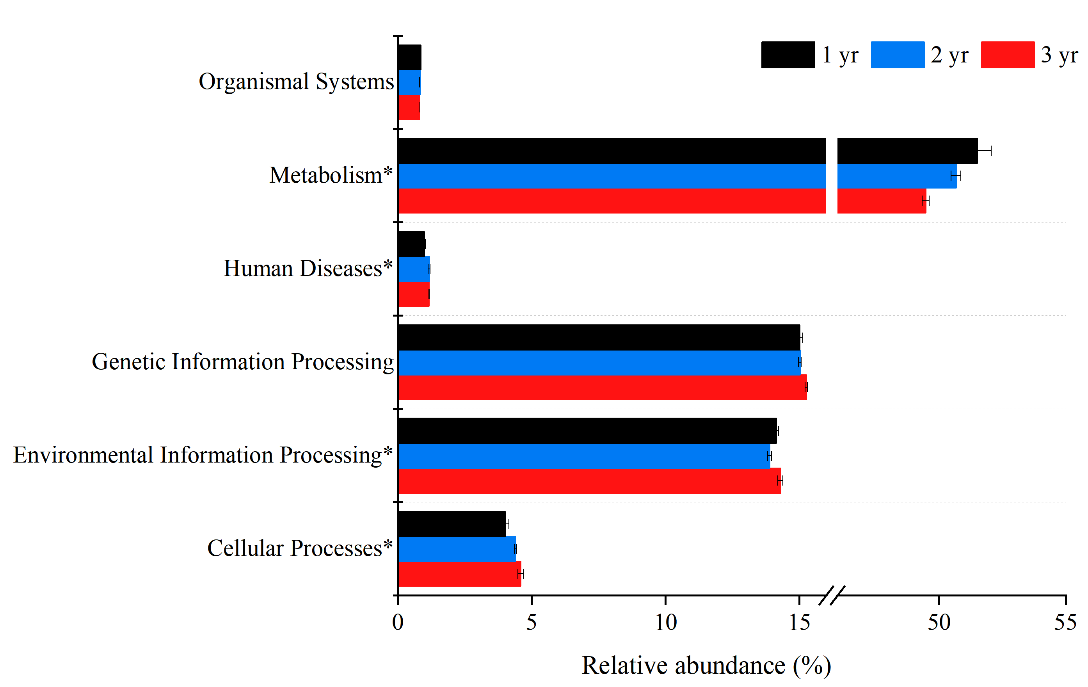
**

**Fig. S4.** Predicted functions of the bacterial communities found in the soil samples derived from different planting years. *Asterisk* above the taxon represents significantly different at *p* < 0.05 according to Duncan’s tests. *Error bars* indicate the standard errors of the means of four replicates.

**
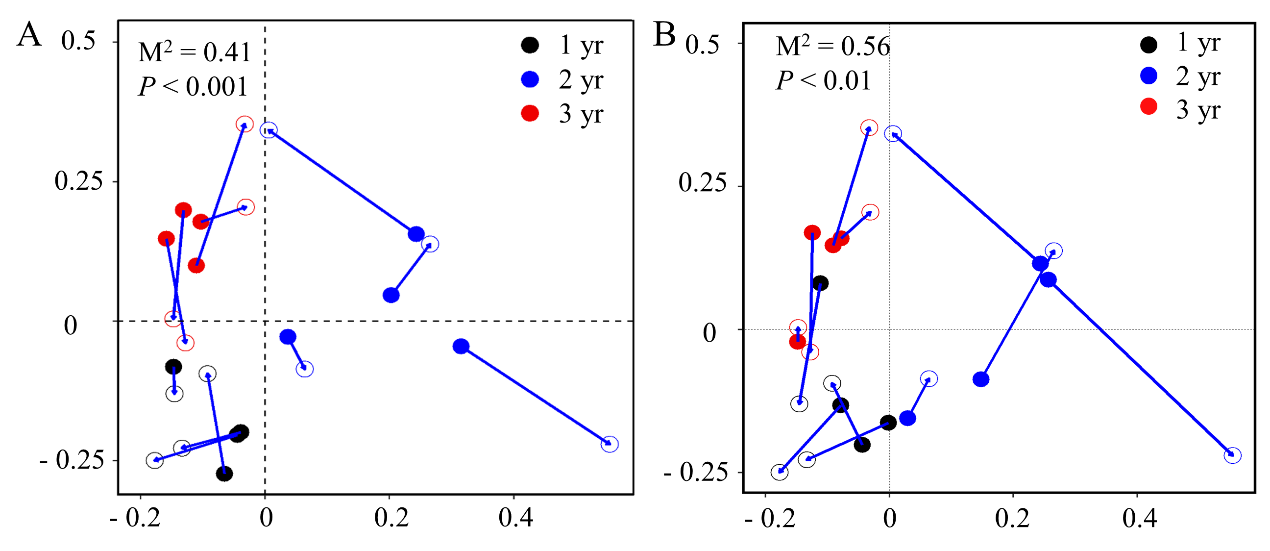
**

**Fig. S5.** Procrustes analyses of PCoA ordination plots between the patterns of phenolic acid profiles and the overall bacterial communities (A) or the beneficial bacterial communities (B), based on the Bray-Curtis distance matrixes.

**Table S1.** The number of unique OTUs for each planting year and overlapped OTUs for every pair of planting years calculated based on the beneficial OTU table.

| Planting year | 1 yr | 2 yr | 3 yr |
| --- | --- | --- | --- |
| 1 yr | ***60*** |  |  |
| 2 yr | *19* | ***10*** |  |
| 3 yr | *11* | *2* | ***5*** |
| Shared OTUs | 53 | 53 | 53 |
| Total OTUs | 143 | 84 | 71 |

Values in bold italics represent unique beneficial OTUs in each planting year, and italics represent overlapped beneficial OTUs between two planting years.

Only the OTUs present in three biological replicates of each planting year were retained for analysis.

**Table S2.** Spearman’s rank-order correlations between activity and diversity of carbon utilization, abundance data of bacteria, and the relative abundance of beneficial bacteria.

| Carbon utilization | Abundance |  | Relative abundance |
| --- | --- | --- | --- |
|  | Bacteria |  | Beneficial bacteria |
| AWCD | 0.41 |  | **-0.66**** |
| McIntosh | **0.60*** |  | **-0.59*** |
| Shannon | 0.42 |  | **-0.74**** |
| Evenness | 0.31 |  | **-0.73**** |

Values in bold indicate significant correlations, **p* < 0.05; ** *p* < 0.01.

**Table S3.** Physicochemical properties of the rhizosphere soil derived from different planting years of root rot-infected Sanqi ginseng

| Planting year | pH | EC (μS/cm^-1^) | SOC (g/kg) | AP (mg/kg) | AK (mg/kg) |
| --- | --- | --- | --- | --- | --- |
| 1 yr | 7.39 ± 0.06 a | 0.19 ± 0.04 ab | 8.49 ± 0.34 a | 6.56 ± 1.48 b | 656.0 ± 44.6 b |
| 2 yr | 6.47 ± 0.46 b | 0.11 ± 0.01 b | 7.26 ± 0.45 a | 4.00 ± 1.15 b | 600.3 ± 79.9 b |
| 3 yr | 7.77 ± 0.06 a | 0.25 ± 0.02 a | 7.71 ± 0.38 a | 12.58 ± 1.39 a | 992.3 ± 81.5 a |

Values are means ± SE (*n* = 4). Different letters indicate significant differences among different rhizosphere soil samples according to Duncan’s test.
